# Effect of Resin Cement Pre-heating on the Push-out Bond Strength of Fiber Post to Root Canal Dentin

**DOI:** 10.15171/joddd.2015.042

**Published:** 2015-12-30

**Authors:** Parnian Alizadeh Oskoee, Ahmad Nooroloyouni, Fatemeh Pornaghi Azar, Jafar Sajjadi Oskoee, Ahmad Pirzadeh Ashraf

**Affiliations:** ^1^Dental and Periodontal Research Center, Tabriz University of Medical Sciences, Tabriz, Iran; ^2^Professor, Department of Operative Dentistry, Faculty of Dentistry, Tabriz University of Medical Sciences, Tabriz, Iran; ^3^Post-graduate Student, Department of Endodontics, Faculty of Dentistry, Tabriz University of Medical Sciences, Tabriz, Iran; ^4^Assistant Professor, Department of Operative Dentistry, Faculty of Dentistry, Tabriz University of Medical Sciences, Tabriz, Iran; ^5^Assistant Professor, Department of Operative Dentistry, Faculty of Dentistry, Tabriz University of Medical Sciences, Tabriz, Iran; ^6^Assistant Professor, Department of Endodontics, Faculty of Dentistry, Tabriz University of Medical Sciences, Tabriz, Iran

**Keywords:** Push-out bond strength, fiber post, resin cement, pre-warming, laboratory research

## Abstract

***Background and aims.*** Various factors influence the interfacial bond between the fiber posts and root canal dentin. The aim of the present study was to evaluate the effect of pre-warming of resin cement on the push-out bond strength of fiber posts to various segments of root canal dentin.

***Materials and methods***. In this in vitro study, 40 single-rooted human premolars were decoronated and underwent root canal treatment along with post space preparation. The samples were randomly divided into two groups: In group 1, Panavia F 2.0 cement was used at room temperature; in group 2, the same cement was warmed to 55‒60°C before mixing. After fiber posts were placed and cemented in the root canals, 3 dentin/post sections (coronal, middle and apical) with a thickness of 3 mm were prepared. A universal testing machine was used to measure push-out bond strength in MPa. Data was analyzed using two-factor ANOVA and a post hoc Tukey test at α=0.05.

***Results. ***The mean value of push-out bond strength was high at room temperature, and the differences in the means of push-out bond strength values between the resin cement temperatures and between different root segments in each temperature were significant (P<0.05).

***Conclusion.*** Pre-warming of Panavia F 2.0 resin cement up to 55-60°C reduced push-out bond strength to root canal dentin. In addition, in each temperature group bond strengths decreased from coronal to apical segments.

## Introduction

Fiber posts were introduced as an alternative to metallic post-and-cores for the restoration of endodontically treated teeth.^1^ Posts with high elasticity and strength are appropriate for such treatments because there is a low risk of displacement and fracture of these posts; fiber-reinforced posts can achieve these requirements in addition to providing good esthetic results.^2^ Since the modulus of elasticity of these posts is close to that of dentin, it is expected that risk of vertical fractures of the root will decrease with the use of these posts.^1,3^ Conventionally, resin cements are used for cementation of fiber posts.^4^

Although vertical root fracture is the most important and a decrease in post retention is the most common reason for the failure of endodontically treated teeth,^5^ another reason for treatment failure in these teeth is the contamination of the root canal through leakage of oral fluids and microorganisms due to inadequate seal and marginal adaptation.^6^ Cementation of a post into the prepared root canal is very important because the cemented post should provide a proper seal along the root canal walls with adequate retention,^6^ which is achieved by increased flowability and deeper penetration of cement into the post space dentin.^7^

It has recently been shown that pre-warming of composite resin decreases its viscosity and film thickness; it also increases composite resin flowability and improves its adaptation with the cavity walls.^8,9^ An increase in temperature improves polymerization kinetics of composite resin in addition to decreasing its viscosity and increasing the degree of conversion^10^ because the motility of free radicals increases under the direct influence of heat on one hand and under the indirect influence of a decrease in viscosity on the other.^11^ An increase in degree of conversion directly influences mechanical properties such as surface roughness, flexural modulus, fracture resistance, shear strength and resistance to abrasion. These characteristics may be clinically important for luting agents, too.^12^ However, dental composites undergo shrinkage during polymerization.^13^ When the degree of conversion of resin monomers increases it is possible that shrinkage and stresses will increase as a result.^13^

Considering the importance of interfacial bond between the resin cement and the root canal dentin, the present study was undertaken to evaluate the effect of pre-warming of Panavia F 2.0 resin cement on push-out bond strength of fiber posts at different segments of root canal. The null hypothesis was that the push-out bond strength of Panavia cement at room temperature is not different from that warmed up to 55-60°C.

## Materials and Methods

Forty human single-rooted mandibular premolars, extracted for orthodontic reasons, were stored in 0.5% chloramine T solution for the purpose of the present study. The teeth were gathered following informed consent, approved by the Deputy Dean of Research at Tabriz Faculty of Dentistry. The working length and root canal morphology were determined radiographically. Teeth with any calcifications or obstructions inside the root canals or with working length exceeding 14 mm were excluded from the study. The teeth were decoronated 1 mm coronal to CEJ using a diamond saw (Isomet, Buchler, Lake Bluff, USA) in a low-speed straight handpiece (Kavo, Germany) under continuous water spray. The root canals were filed manually to the full working length using the step-back technique and #15 to #40 K-files (MANI, Tochigi, Japan) with a master apical file of #30; and flared using #2 to #4 Gates-Glidden drills (MANI). After each instrumentation step, the canals were irrigated with normal saline solution. The canals were dried with paper points (PT Dent, USA) and obturated with gutta-percha and AH26 sealer (De Trey, Zurich, Switzerland) using lateral condensation technique.

Post space was prepared 24 hours after completion of endodontic procedures. The root canals were prepared up to 10 mm using #2 and #3 Peeso reamers (MANI) and the special drills provided by the manufacturer for MATCHPOST #1/4 (RTD, St Igreve, France). MATCHPOST is a radiopaque translucent fiber post. Its diameter is 1.42 mm in the coronal part and 0.77 mm in the apical end. It is 19 mm in length with the first 15 mm parallel and a homogeneous taper at its 4-mm end. The roots were placed in a mold of condensation silicone impression material with a putty consistency (Speedex, Coltene, Switzerland) and divided into two groups.

The posts were cleaned with 70% ethanol, based on manufacturer's instructions and dried with a compressed air current. In group 1, Panavia F 2.0 resin cement was used at room temperature based on manufacturer's instructions: at first, an equal amount of ED-Prime II was mixed on a mixing dish for 30 seconds and was placed on the clean surface of the fiber post and the root canal using a microbrush. After 60 seconds, an equal amount of the two syringes of the cement, which had been mixed for 20 seconds on a glass slab, was placed within the canal and the fiber post was seated in place using finger pressure. The resin cement was light-cured for 20 seconds using a light-curing unit (Astralis 7, Ivoclar, Vivadent, Liechtenstein) at a light intensity of 750 mW/cm^2^. The light-curing tip was placed at the canal orifice.

The light intensity of the light-curing unit was measured and controlled during the curing process using a light meter (Coltene, Whaledent, Switzerland).

In group 2 the procedural steps were similar to those in group 1 except that before application of the resin cement, the cement was immersed for 12 minutes in a warm thermostatically-controlled water bath with a temperature range of 55-60°C (TELEDYNE HANAU, Buffalo, N.Y, USA).

The samples were retrieved from the silicone molds and immersed in distilled water for 24 hours. Then the samples were fixed on the lab cutting instrument using sticky wax and were horizontally cut perpendicular to the long axis of the tooth under water spray. Each specimen yielded three post/dentin sections (cervical, middle and apical) with a thickness of 3 mm.

Therefore, each group yielded 60 sections, which consisted of 3 sections equally divided for cervical, middle and apical thirds. The actual length of each fiber post segment in each section was measured using digital calipers (Mitutoya, Tokyo, Japan) with an accuracy of 0.01 mm. The push-out test was carried out using the special jig of the push-out test.

The samples were fixed on the push-out jig using cyanoacrylate glue. Therefore, the cervical part was placed toward the jig and the post was placed at the center of the space between the metallic supports. The cylindrical piston with a diameter of 0.7 mm was exactly positioned at the post center, avoiding contact with the post periphery. The force was applied in an apico-cervical direction at a strain rate of 0.5 mm/min using a universal testing machine (Hounsfield Test Equipment, model: H5K-S, England).

The maximum force at the point of the extrusion of the post segment from the test sample was recorded in Newton.

The push-out bond strength was calculated in MPa using the following formula:

$$\text{Bond Strength}=\;\frac{\text{Force (N)}}{\text{Sample surface area (A)}}$$

The surface area (A) was calculated by the formula: A=3.14 [n (r_1_+r_2_)], in which r_1_ represents the radius of the cervical area of the post, r_2_ represents the radius of the apical area of the post and n can be calculated using the following formula: n=√(r_1_-r_2_)^2^+h, where h is the thickness of the slice in millimeter.^14^

Data was analyzed using two-way ANOVA and post hoc Tukey test at significance level of α=0.05.

## Results

**Figure 1.**
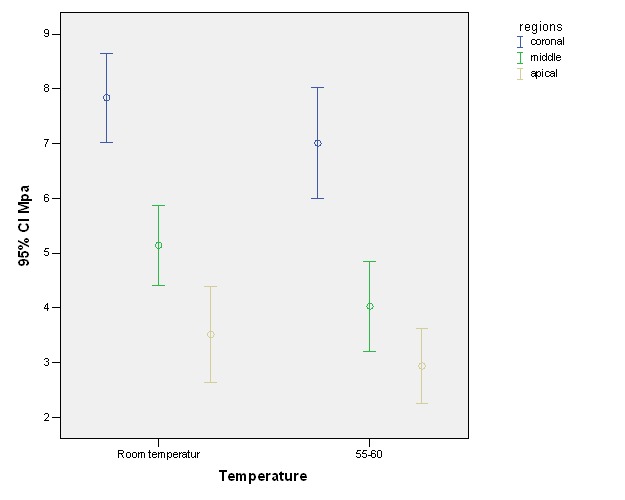
Error Bar of the bond strengths in different root segments in two different temperatures.

Table 1 presents means and standard deviations of push-out bond strength values in the study groups. Based on the results, the mean value of push-out bond strength at room temperature was higher than that at 55-60°C (5.5±0.22 vs. 4.65±0.22). This difference was significant according to the results of two-way ANOVA (P=0.01). In addition, there were significant differences in the means of push-out bond strength values between the different root segments (P<0.001). However, the interactive effect of the two variables (temperature and different root segments) was not significant (P=0.79). Two-by-two comparisons of different root segments at room temperature and 50-60°C, separately, with the use of a post hoc Tukey test revealed significant differences between the coronal, middle and apical thirds of the root at room temperature: coronal and middle third (P<0.001); middle and apical third (P=0.01); coronal and apical third (P<0.001) (Figure 1). At 55-60°C, the differences between the coronal and middle thirds and between the coronal and apical thirds were significant (P<0.001); however, there were no significant differences between the middle and apical thirds (P=0.14) (Figure 1).

**Table 1:** Means ± standard deviations (SD) of bond strength values in the study groups

**Temperature**	**Region**	**Mean**	**SD**	**Std. error**	**Minimum**	**Maximum**
**Room** **Temperature**	Coronal	7.84 (a)	1.73	0.38	4.24	11.51
	Middle	5.14 (b)	1.57	0.35	1.71	7.74
	Apical	3.51 (c)	1.88	0.42	0.78	7.18
**55-60°C**	Coronal	7.00 (a)	2.15	0.48	3.37	11.65
	Middle	4.02 (b)	1.75	0.39	0.56	7.21
	Apical	2.93 (b)	1.45	0.32	0.88	5.72

## Discussion

Retention of a fiber post within the root canal depends not only on the bond strength between the post and the luting agent but also on the bond strength between the luting agent and the post space dentin.^14^ Scanning electron microscopy studies have shown a proper bond between the matrix of the posts and resin luting agent;^15^ however, debonding along dentin-resin luting agent interface has been identified as the most frequently encountered mode of failure.^16^ The bond strength at post-cement and cement-dentin interfaces is influenced by various factors, including the reaction between dentin and the luting agent, polymerization rate and stresses resulting from polymerization shrinkage of the resin luting agent, presence of the remnants of endodontic sealer or gutta-percha, configuration of the root canal, and differences in density and orientation of dentinal tubules in different root segments.^17^ Regarding the ever-increasing use of fiber posts and the importance of establishing a proper bond between the fiber post and root canal dentin for the success of treatment, evaluation of bond strength at post-cement and cement-dentin interfaces is of utmost significance. Bond strength might be evaluated by various techniques. The push-out bond strength test is an appropriate test to evaluate the bond strength between endodontic posts and intra-canal dentin. In the push-out technique, failure occurs along the post-cement and cement-dentin interfaces, which is similar to clinical situations.^18^ According to Goracci et al^19^ it appears the push-out test is more reliable than microtensile technique. Therefore, in the present study, the effect of pre-heating of resin cement before mixing on push-out bond strength of fiber post to intracanal dentin was evaluated. Based on the results, the bond strength of fiber post to intracanal dentin significantly decreased when the resin cement was pre-warmed. Pre-warming technique for restorative composite resins has been recommended due to its positive effects on decreasing viscosity, improving flow, decreasing film thickness and increasing monomer conversion rate of composite resins in addition to its clinical advantages of facilitating material placement and its adaptation with cavity walls.^11^ Cantoro et al^20^ evaluated the effect of minor temperature changes in resin cements and based on the results recommended that resin cements stored in refrigerator should be warmed at least to room temperature before use. Of course, it should be pointed out that various cements with different adhesion strategies exhibit different behaviors when they are pre-warmed.^20^ Although the effect of temperature on the degree of conversion and the flowability of resin composites cannot be denied, it seems that the temperature of cement drops during mixing and dispensing processes.^7^ Therefore, an increase in the degree of conversion and polymerization shrinkage of pre-heated resin will be lower than what we have expected.^9^

Dimethacrylate-based composite resins are polymerized through a free radical polymerization reaction.^21^ The amount of polymerization in composite resins is expressed by degree of conversion which is defined as the percentage of conversion of C=C monomer bonds to C-C polymer bonds.^11^ Degree of conversion influences the mechanical and physical properties of the polymer.^22^ Surface roughness, flexural strength, flexural modulus, fracture resistance, diametral tensile strength and abrasion resistance increase with an increase in degree of conversion.^23^ Recent studies have shown that curing of composite resins at higher temperatures results in higher conversion rates.^11^ Composite resins undergo shrinkage during polymerization procedures;^24^ in addition to physical and mechanical properties, the amount of shrinkage and shrinkage strain of composite resins change proportional to degree of conversion.^25^ In other words, polymerization shrinkage and the resultant stresses increase with an increase in conversion rate.^26^

When polymerization shrinkage is limited by adhesion to cavity walls, in addition to substrate compliance, another factor which should be considered for the release of polymerization stresses is configuration factor (C-factor), which is defined as the ratio of bonded surface to unbonded surface of the composite resin.^27^ C-factor and polymerization shrinkage stress are inversely related.^28^ The root canal has a high C-factor.^29^ Since the thermal expansion coefficient of composite resins is 6-8 times greater than that of surrounding tooth structures^30^ it appears when the resin cement is pre-heated up to 55-60°C, high C-factor of the root canal neutralizes the increase in flowability and marginal adaptation and the decrease in film thickness, producing an unfavorable condition for bonding of the fiber post to the root canal dentin; therefore, a decrease in push-out bond strength was observed. However, Cantoro et al^20^ reported high bond strength values when Panavia F 2.0 cement was pre-warmed. Of course, it should be pointed out that in this study the resin cement was bonded to the smooth dentin surface and the effect of C-factor was not significant.

Another finding of the present study was the higher mean of push-out bond strength of fiber post in the coronal third compared to the middle and apical thirds, with the lowest bond strength in the apical third of the root canal. Although Bitter demonstrated that the apical bond strength of Panavia F cement was higher;^31^ and in a study carried out by Oskoee et al,^14^ it was shown that the self-etch technique did not result in a significant difference in the push-out bond strength in different root segments, the results of the present study in relation to the effect of various root segments on bond strength are consistent with the results of previous studies.^18,32,33^ Based on SEM studies, adhesive bonding to the root canal dentin was mainly attributed to the formation of resin tags.^34^ The density of dentinal tubules and the number and length of resin tags significantly decrease in the cervico-apical direction.^14^ On the other hand, problems related to manipulation and access to narrow and terminal parts of the apical region of root canal and the probable presence of the smear layer or gutta-percha remnants^35^ might interfere with thorough penetration of the adhesive and resin cement into the dentinal tubules, compromising the bond strength in the apical regions of the root canal.

Finally, it should be pointed out that despite the importance of in vitro studies, it is difficult to extend the results to clinical situations. In addition, considering the varieties and different functions of resin cements and the importance of their other properties, it is suggested that the effect of pre-warming of cements on their physical and mechanical properties and also their shelf life be evaluated in situations as similar to oral cavity conditions as possible. Besides, precise evaluation of cement-dentin interface in the above-mentioned conditions might furnish useful data.

## Conclusion

Under the limitations of the present study it can be concluded that pre-warming of Panavia F 2.0 resin cement up to 55-60°C significantly decreases the bond strength of cement to root canal dentin. Also in each temperature group bond strengths decreased from coronal to apical segments.

## Acknowledgments

This research was carried out by the financial support from the deputy Dean of Research at Tabriz University of Medical Sciences. The authors also thanks Dr. M. Abdolrahimi, DDS, who edited the English manuscript of this article.
